# Carcinoma penis: How late can inguinal nodal metastases occur?

**DOI:** 10.4103/0970-1591.33735

**Published:** 2007

**Authors:** Anil Kamath, T. B. Yuvaraja, H. B. Tongaonkar, S. Kane

**Affiliations:** Department of Genitourinary Oncology, Tata Memorial Hospital, Parel, Mumbai, India; * Department of Pathology, Tata Memorial Hospital, Parel, Mumbai, India

**Keywords:** Carcinoma penis, inguinal nodes, recurrence

## Abstract

Inguinal nodal metastasis is the single most important prognostic factor for survival in a patient with carcinoma penis. In patients without inguinal lymph nodal metastasis at presentation, options include close surveillance or prophylactic inguinal lymph nodal dissection. The majority of patients on surveillance who develop inguinal nodal metastases do so within two to three years of treatment of the primary. Here we report a case who developed inguinal nodal metastasis 10 years after the treatment of primary. This raises questions about the natural history and biology of the disease, the optimum surveillance and whether a patient of carcinoma penis can ever be considered risk-free for metastasis.

## INTRODUCTION

Inguinal nodal metastasis is the most important prognostic factor for survival in a patient with carcinoma penis. While the standard treatment for node-positive disease is a groin nodal dissection, the management of clinically negative groin is controversial. In patients with T2-T4 disease incidence of occult nodal metastases is high and prophylactic nodal dissection is recommended by many although there is no Level 1 evidence supporting this practice. Other options include wait and watch policy where patients are closely observed and lymph node dissection is done at relapse. The optimum surveillance has not been clearly defined. Most studies addressing the issue have examined patients once in three months for the first two years and once in six months for the next two to three years and then yearly thereafter. This is based on the premise that most inguinal metastases occur within the first two to three years. There have been no reported cases of inguinal recurrences beyond five years in any large series. Here we report the case of one of our patients who had inguinal nodal recurrence 10 years after treatment for the primary. This shows that a few patients can be at risk of inguinal metastases even after the initial period of active surveillance.

## CASE REPORT

**Figure 1:**
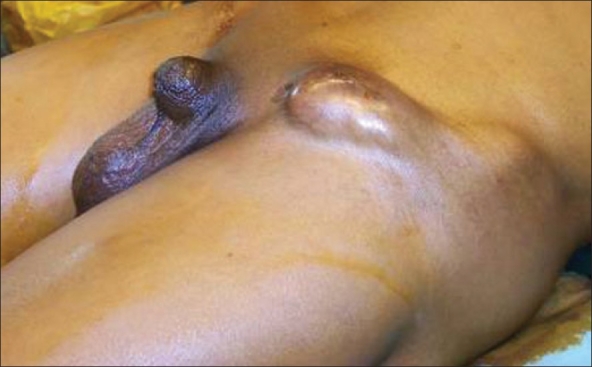
Operated carcinoma penis with inguinal nodes

A 53-year-old man presented to us with left inguinal nodal mass of four months duration. The patient was diagnosed to have carcinoma of the penis at our hospital in 1996 and had undergone a partial penectomy [Figure 1]. Histopathology of the penectomy specimen showed a moderately differentiated squamous carcinoma infiltrating the corpora cavernosa (pT2 cN0). Cut margins were free. There was no lymphovascular invasion. As there were no palpable groin nodes at the time of penectomy, surveillance policy was adopted. The patient had subsequently come for regular follow-up for five years. Physical examination at the time of follow-up was normal with no evidence of recurrence either at the primary site or in the groin after which patient failed to follow up.

On physical examination the partial penectomy site was healthy with no evidence of recurrence. There was a 6×5 cm mass in the left groin with inflammation of the overlying skin. The right groin and penile stump were normal. The rest of the physical examination and metastatic workup was normal.

**Figure 2:**
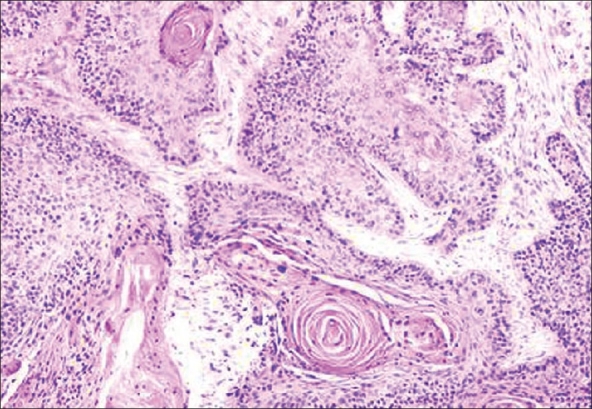
Histopathology of inguinal nodes showing squamous carcinoma

A fine needle aspiration cytology of the groin nodes confirmed metastatic squamous carcinoma cells. Ultrasound abdomen did not reveal any iliac nodes. The patient underwent left ilio-inguinal lymph nodal dissection with anterolateral thigh flap reconstruction. Histopathology revealed metastatic squamous carcinoma [Figure 2] with necrosis, calcification and fibrosis and peri nodal extension of tumor and was infiltrating the overlying skin. The iliac nodes were negative for metastases. The patient received adjuvant chemotherapy in the form of gemcitabine and cisplatin and was disease-free six months after completion of chemotherapy as on December 2006.

## DISCUSSION

Management of clinically negative groin in patients with carcinoma of the penis has been a highly contentious issue. The incidence of metachronous inguinal metastases in patients with T1 lesions is low. In studies by Solsona[1] and Villavicencio[2] the incidence of early stage tumors has been associated with a 4-14% incidence of lymph nodal metastases. This has led clinicians to offer close surveillance as a reasonable option to patients with T1 carcinoma penis.

The question as to what constitutes optimum surveillance has not been addressed in any randomized trials. Campbell[3] recommends three-monthly follow-up for the first two years, four-monthly follow-up in the third year, six-monthly follow-up in the fourth year and then annual follow-up from fifth year onwards for the low-risk patients (Tis, Ta, T1 Grade 1-2 with no lymphovascular invasion). The recommended follow-up for high-risk cases (T2,3 Grade 3, vascular invasion) is two-monthly for the first two years, three-monthly follow-up in the third year and six-monthly follow-up in the fourth year and then annually. The surveillance is more intense in the first two to three years as most inguinal metastases occur within this period.

In a study from Netherlands by Horenblas[4] involving 110 patients, all regional recurrences developed within two years of primary treatment. In another nonrandomized study by Ravi[5] 258 patients with invasive penile cancer and negative groin nodes were observed. All patients in this group who developed groin recurrences did so within 18 months of surgery for the primary tumor. The outcomes of initial surveillance of patients with invasive carcinoma penis with negative nodes were studied in detail by Theodorescu D.[6] Of the 42 patients with T1-3 penile cancers 26 (62%) developed inguinal nodal recurrences during follow-up. Fifty per cent of those who developed recurrences did so within 1.4 years and 75% within 2.8 years. The authors of this study used the Kaplan Meier method to estimate the interval to inguinal recurrence. The node recurrence-free possibility at six years was zero which meant that at the rate at which patients were developing nodal recurrence all should have developed recurrence by six years of follow-up.

The present case raises a few questions about the natural history of carcinoma penis. Firstly, most studies use a five-year disease-free survival cutoff and such patients with delayed presentation are likely to be falsely assumed to be cured in most studies. Second is the question of optimum surveillance. The present case raises the need for continued surveillance beyond five years.
